# Editorial: Supramolecular Nucleic Acid Chemistry

**DOI:** 10.3389/fchem.2020.00749

**Published:** 2020-08-31

**Authors:** James H. R. Tucker, Janarthanan Jayawickramarajah

**Affiliations:** ^1^School of Chemistry, University of Birmingham, Birmingham, United Kingdom; ^2^Department of Chemistry, Tulane University, New Orleans, LA, United States

**Keywords:** supramolecular, nucleic acids, DNA, self-assembly, molecular assemblies

The reversible and non-covalent nature of the interactions governing the structure and binding properties of nucleic acids mean that the fields of supramolecular chemistry and nucleic acid chemistry are closely intertwined. Indeed, the high fidelity and programmable molecular recognition properties of DNA have long been the source of inspiration to many a supramolecular chemist in designing molecular assemblies that are both discrete in shape and dynamic in nature. However at the same time, researchers working in the field of nucleic acids can draw inspiration and rationale from concepts and approaches that are rooted in supramolecular chemistry. For example, many themes central to supramolecular chemistry such as sensing, switching, self-assembly, catalysis, and molecular motion can be successfully applied to nucleic acid systems. This Research Topic is a compilation of 12 original papers from 50 authors and nine different countries on nucleic acid chemistry that highlight the synergies and links with supramolecular chemistry.

The collection includes two topical reviews. Firstly there is an overview by Takezawa and Shionoya of DNA three-way-junction motifs that are bridged by interstrand metal complexes; such structures could lead to new, tuneable metal-responsive supramolecular architectures based on nucleic acids. The second by Michel et al. gives a broad and detailed account of the state-of-the-art on environment-sensitive fluorescent nucleic acid probes of relevance to sensing and cell imaging applications. Following on with the sensing theme, an electrochemical approach to detecting DNA is presented by Goodchild et al., who report a new surface-bound probe with a redox tag that can discriminate between closely-related sequences, down to changes at the single base level. Another study on tagged DNA comes from Burns et al. who report a novel molecular ruler system comprised of a series of DNA strands modified in two positions with metal-bound and metal-free porphyrin groups that enable the detection and monitoring of small changes in DNA structure.

Various non-canonical DNA structural motifs are the theme of several articles in this collection, including three-way junctions (vide supra), G-quadruplexes, and i-motifs. A report by Punt et al. describes how the covalent incorporation of different ligands into DNA and their binding of d block metals impart significant changes on G-quadruplex stability, with clear implications for the controlled design of functional metal-containing nucleic acid complexes. Molecules that interact with G-quadruplexes are the focus for Ranjan et al. and in particular dual-binding ligands with both groove binding and intercalating moieties that form strong and selective complexes with these structures. The i-motif and the possibility of it playing a role in biological processes is currently a topic of considerable interest in the field of nucleic acid chemistry. An important contribution to the discussion is the report by Abdelhamid and Waller, who show how kinetic effects such as annealing and equilibration times are important factors in determining the longevity at neutral pH and ambient temperature of an i-motif structure formed from a sequence in the human telomeric region. Meanwhile Pages et al. show that the length and position of thymine loops within an i-motif forming sequence affects its structure as well as the extent of its stabilization by enantiomers of an octahedral Ru(II) complex.

How genetic information might have been transferred prebiotically on earth in the absence of enzymes continues to fascinate chemists. An intriguing paper by Núñez-Pertíñez and Wilks considers the possibility of deep-eutectic solvents (DESs) as stabilizing media for prebiotic reactions, using nucleic acid-templated peptide synthesis as their model. Rather different DNA-templated systems are considered by Fritz and Wagenknecht. These authors reveal the important structural parameters required to form DNA-templated assemblies of perylene-nucleoside derivatives in aqueous solutions, which includes having an ethynylene linker between the nucleobase and the chromophore. The supramolecular assembly theme continues in work reported by Atchimnaidu et al., who prepared β-CD-functionalised DNA assemblies for the binding of star-shaped adamantyl PEG polymers, resulting in the formation of non-covalent crosslinked nanoparticles for capturing both hydrophobic and hydrophilic micropollutants in water. Finally, rather different cross-linked systems are reported by Cadoni et al., who demonstrate an effective method for immobilizing PNA strands onto maleimide-functionalized gold nanoparticles, via a double exchange Diels-Alder cycloaddition reaction. These immobilized strands retain their ability to interact with DNA, with the resulting duplexes able to be released through a retro-Diels-Alder reaction.

In summary, this Research Topic has highlighted how the fields of nucleic acids and supramolecular chemistry can be imbricated to generate hybrid systems (such as assemblies that harness canonical/non-canonical base-pairing as well as synthetic metal-ligand, host-guest, and aromatic stacking interactions) of fundamental scientific interest. Further, the Topic also illustrates the potential for developing exciting function, including detecting specific DNA sequences, binding to folded DNA structures for biomedical applications, and generating assemblies capable of environmental remediation. Although just a snap-shot of the diversity of research directions being pursued in Supramolecular Nucleic Acid Chemistry—it is clear that the field shows much promise in not only generating increasingly complex systems but also in addressing topical issues plaguing modern society.

## Author Contributions

All authors listed have made a substantial, direct and intellectual contribution to the work, and approved it for publication.

## Conflict of Interest

The authors declare that the research was conducted in the absence of any commercial or financial relationships that could be construed as a potential conflict of interest.

